# From ADHD Diagnosis to Meaning: Does Grief Theory Enhance Our Understanding of Narrative Reconstruction?

**DOI:** 10.3390/brainsci15101045

**Published:** 2025-09-25

**Authors:** Kate Carr-Fanning, Aoife M. Lynam, Tom Nicholson, Conor McGuckin

**Affiliations:** 1 School of Education, University College Dublin (UCD), D04 V1W8 Dublin, Ireland; 2 School of Education, Trinity College Dublin, D02 PN40 Dublin, Ireland; aolynam@tcd.ie (A.M.L.); conormcguckin@gmail.com (C.M.); 3 Department of Nursing, Midwifery & Health, University of Northumbria, Newcastle upon Tyne NE1 8ST, UK; t.nicholson@northumria.ac.uk

**Keywords:** grief, loss, diagnosis, disability, neurodiversity, parents, adults, adjustment, psychosocial adaptation

## Abstract

While having or raising a child with a neurodivergence can be rewarding and life-enhancing, a diagnosis may trigger stress as people adapt to and navigate a new “assumptive world”. The purpose of this paper is to draw on the Attention Deficit Hyperactivity Disorder (ADHD) literature and explore it using grief theory as a conceptual framework, so as to understand parents’ and adults’ potential reactions to a diagnosis of ADHD. A narrative review of the ADHD literature suggests that adapting to a diagnosis can be understood as a process, which can occur in decisions to seek, and in reactions to, a diagnosis. For some, diagnostic work can be abrupt and unexpected, a life-changing moment when one receives an initial diagnosis. For others, diagnostic work begins pre-diagnosis. Either way, the diagnostic process requires an adjustment. This adaptation, like grief, can be ongoing and can involve a psychosocial transition. Rather than traditional models of grief (e.g., Freud and Kübler-Ross), contemporary views of grief such as the Dual Process Model and Meaning Making enable us to understand the meaning-making that is central to diagnostics work. Pre-diagnostically, people must become aware that something is different than previous expectations, in order to seek a diagnosis. Exposure to ADHD knowledge and stigma is influential pre-diagnostically. Post-diagnostically, people encounter a range of emotions which they need to process, such as grief for the past, making decisions (e.g., treatment), worries for the future, and reconstructing identity. The neurodiversity paradigm poses opportunities and challenges for people within this meaning-making process. This paper identifies implications for practice, particularly around pre- and post-diagnostic support, and directions for future research.

## 1. Introduction

Diagnosis typically represents a point of psychosocial transition for individuals and their families. This paper draws on grief theory, with a particular focus on psychosocial transition [1], rather than traditional models [2,3], to explore how individuals adjust [4,5]. People may experience stress, even permanent stress, following a diagnosis [6], including feeling intense distress akin to grief [7,8]. Sinclair [9] acknowledged, in “Don’t Mourn for Us”, that parents may feel such grief due to their “shattered expectations” that they have for their child. However, some diagnoses may be more stressful for parents to receive than others (e.g., Larkin et al. [10]), and this stress, like grief, can be ongoing and involve normative adaptive stress [11].

Evidence suggests that parents’ reactions are markers of the parent and child’s well-being, which may impact family functioning. Parents who resolve themselves to a diagnosis may be more securely attached to their children [12], whereas those who struggle to accept their child’s diagnosis may be more detached, harbour unrealistic and unbalanced beliefs, and experience confusion [13]. Different factors are suggested to be involved in the resolution of a diagnosis, such as resilience [14], stress processing [15], parental self-efficacy [16,17,18], and coping strategies [19]. Conversely, a lack of coping skills can lead to negative outcomes for parents/families, such as depression, isolation, and spousal problems [20,21].

It is difficult to estimate ADHD prevalence. Research suggests diagnostic rates for ADHD are 7.2% in childhood [22], and Kooij et al. [23] estimate that 2–4% of adults have ADHD. However, many more (especially adults) may choose to self-identify as ADHD without a diagnosis and so are not captured within the prevalence figures. The Irish Health Survey [24] reported that 18.7% of the participating adults (N = 5101) identified as neurodivergent, with most self-identifying as ADHD (9%), rather than actually having received a diagnosis (2%). We cannot know how many of these are languishing on waiting lists or choosing to live without a diagnosis. Research suggests children [25] and adults [26] report waiting years to receive (and have to “fight for”) a diagnosis. There will also be people who self-identify but who do not meet the diagnostic criteria for ADHD. Therefore, we can assume that for at least some, diagnostic work begins pre-diagnosis, while for others, it may occur post-diagnosis.

This paper explores whether and how grief theory provides a framework to understand how parents and adults adjust to a potential or actual diagnosis of ADHD, to develop a deeper understanding of how they can process this change at different stages, pre- and post-diagnosis. We begin with the theoretical framework provided by grief theory. In line with contemporary grief literature, we do not adopt a clinical or pathologising stance on grief; rather, we view it as a normative process of adaptation and meaning reconstruction, distinct from older stage-based models that emphasised resolution or “acceptance” as an endpoint [5,27]. Then, we move on to consider the meaning-making process in diagnostic work for parents, before exploring how adults make sense of their own diagnosis. Diagnostic work with neurodivergences is unique, and with the advent of the neuro-affirmative paradigm, it is changing. For some, the process of grief and acceptance might begin/occur during the pre-diagnosis stage, while for others it can be abrupt, post-diagnosis. This paper will critically explore the adaptation process, to understand the nuances within the diagnostic journeys of parents and adults, and how they make sense of these unique experiences.

## 2. Grief Theory

By its very nature, grief can defy definition due to individual responses, which include variables such as, for example, development, psychological well-being, and circumstances (e.g., type, personal, situational). Due to the complexity and intangibility of the concept of grief, there have been various schools of thought surrounding the concept. While definitions vary, grief is widely understood as a cognitive and emotional process enabling individuals to confront loss, reconstruct meaning, and adapt to a changed reality [4,5].

### 2.1. Traditional Approaches to Grief: Stage and Task-Based Models

Over the past century, theoretical understandings of grief have developed considerably, reflecting wider shifts in psychology, culture, and empirical inquiry. Freud’s [2] seminal psychoanalytic essay “Mourning and Melancholia” conceptualised grief as a process of “decathexis,” in which the bereaved gradually withdraw libidinal energy from the lost attachment (i.e., the deceased) and reinvest it elsewhere. This intrapsychic model positioned detachment as the central task of mourning. By contrast, mid-twentieth-century discourse was dominated by Kübler-Ross’s [3] stage theory, which proposed a universal five-stage sequence of denial, anger, bargaining, and depression, before finally reaching acceptance. Although originally derived from work with terminally ill patients, it became widely applied to bereavement, and has been regularly popularised in public culture (e.g., “The Simpsons”, *Bridget Jones*, *Mad About the Boy*). Its enduring influence notwithstanding, the model has been critiqued for its linearity and its limited capacity to capture the heterogeneity of individual grief trajectories [4]. Subsequent theoretical advances emphasised adaptation and reconstruction rather than staged progression. Collectively, these perspectives illustrate the movement from prescriptive and universalising accounts toward an understanding that grief encompasses variability, identity disruption, and meaning reconstruction. A child’s diagnosis can represent such a transition, challenging assumptions of “what should be” and requiring adaptation to new identities and roles. Parkes [1] describes grief as a disruption in one’s “Assumptive World”, a framework of expectations about life, necessitating the construction of a new one, a process Attig [28] terms “relearning the world.” As suggested by Parkes [1], creating a new “assumptive world” is one of the challenges faced by the grieving. Parkes [1] notes that we all live in a world where we “assume” life will carry on as it always has, with its common first-order changes; however, the assumptive world can be shattered when a second-order change occurs. A diagnosis is, arguably, a significant second-order change.

For parents receiving a child’s diagnosis, this entails reinterpreting expectations, roles, and futures within a newly reconstructed assumptive world. When applied to the context of a child’s diagnosis, these grief theories suggest that parents may experience the event as a psychosocial disruption rather than solely a clinical matter.

### 2.2. Contemporary Approaches to Grief: Relational and Constructivist Perspectives

More contemporary grief theory shows how The Dual Process Model [4] advances a dynamic view, proposing oscillation between loss-oriented coping (focusing on grief and the pre-loss world) and restoration-oriented coping (addressing secondary changes, adapting to new roles). Through their Dual Process Model, Stroebe and Schut [4] conceptualise grief as confronting the “before and after” of an event, focusing on memories, and working toward rebuilding a new life. Earlier stage-based theories of grief (e.g., [3]) often framed “acceptance” as the final stage or necessary outcome of the grieving process. More contemporary perspectives, however, have problematised this framing, noting that grief does not require resolution or closure, and that pathologising those who do not reach “acceptance” is unhelpful [5,27]. Instead, newer models emphasise normalising grief as a continuing experience and recognising that many individuals maintain ongoing bonds with the deceased, integrating loss into their lives rather than relinquishing it. A parallel can be drawn to adapting to an ADHD diagnosis, where the task is not to “accept” the diagnosis in a linear or final sense, but to engage in meaning-making and identity reconstruction. The Dual Process Model [4] is instructive here, as it highlights how individuals oscillate between confronting the losses associated with the diagnosis (e.g., missed opportunities, altered expectations) and engaging in restorative coping directed toward the future. In this way, the diagnosis becomes incorporated into a revised life narrative, supporting adaptation without requiring a definitive endpoint of acceptance.

According to Attig [28], experiences of loss necessitate a reconfiguration of one’s social world, as the bereaved must integrate the absence into their lives and confront the associated suffering. This reconstruction may involve either efforts to soften the intensity of distress or processes of accommodation that allow the individual to live with it. Within this framework, meaning-making is the central task, as adaptation depends on the capacity to re-establish coherence and continuity in the wake of disruption. Meaning reconstruction is considered successful when the bereaved formulate a new narrative that sustains a revised assumptive worldview [1,4]. This narrative orientation aligns with social constructivist perspectives [29], which hold that perceptions of reality are shaped through individual experience and interpretive frameworks. The iterative act of narrating loss enables grievers to situate the event within their life story, to accept its finality, and to recognise the transformation of their world that results. Conversely, Neimeyer [5] argues that unresolved grief is often characterised by a failure to generate such a coherent narrative that accommodates the meaning of loss. Similarly, for a parent, a family, or an adult who is coming to terms with a diagnosis, creating a new narrative around their own lives and/or for their child’s condition may be necessary in order to understand and adapt to their new world that has been reconstructed. Adjustment to a diagnosis depends on reconstructing a coherent individual and family narrative that integrates the diagnosis into a renewed sense of identity and purpose, fostering resilience and hope. Together, these grief frameworks underscore that a child’s diagnosis can be a life-altering transition that demands a psychosocial transition, emotional processing, cognitive reorganisation, and the creation of new meaning.

## 3. Why ADHD and Grief Work?

ADHD is a neurodevelopmental condition characterised by “symptoms” of hyperactivity, impulsivity and/or inattention (DSM5). While historically viewed as a “behaviour problem”, recently a more nuanced understanding of ADHD includes recognition for the diversity of presentation across individuals, including emotional regulation and executive functioning [30]. While once considered only a disorder, the neurodiversity movement, driven by advocates and the lay community, has championed a paradigm of ADHD as a neurodevelopmental “difference” in how people think, learn, process information, and interact with the world. These differences are viewed and celebrated as a normal part of human variation—just one more aspect of diversity, similar to race/ethnicity, hair colour, gender, etcetera [31].

Diagnostic rates are rising for both children [32] and adults [33]. Therefore, there is a need to understand how people navigate the process of seeking and adjusting to receiving a diagnosis. ADHD represents a good case study to explore adjusting to a diagnosis for several reasons. First, grief theory implies loss and coming to terms with that loss. However, with the advent of neurodiversity, receiving a diagnosis could be a positive, life-affirming experience. Therefore, we must be cautious not to reinforce deficit-perspectives, because the grief model could legitimise the “broken” narrative, where something is “wrong” with people with ADHD. At the same time, we need to be cautious about imposing a strengths-based perspective, which may undermine people’s actual lived experiences. In this way, the psychosocial transition and meaning-making frameworks around negotiated identities and world views of modern grief theory provide useful frameworks to explore how people navigate these different lenses. ADHD is also unique, because as we shall see, grief and meaning-making may occur before receiving a diagnosis, and people with ADHD may self-identify and never receive a formal diagnosis. Indeed, the literature reviewed here is based on those who have successfully received a diagnosis, and so we cannot know other people’s experiences. Finally, since ADHD is being diagnosed in childhood and adulthood, an understanding of grief, loss, and meaning-making for parents and adults is important. We use the term “parents” to refer to those in parenting roles; caregivers who may or may not be biologically related to the children they parent. This paper draws on grief theory and the ADHD literature to explore the process of meaning-making, both before and after receiving a diagnosis, from the perspective of parents with children with ADHD and adults with ADHD, to explore how people make sense of a diagnosis and reconstruct assumptive worlds.

## 4. Diagnosis in Childhood: Parental Grief Work

We begin by exploring diagnosis work and grief work through the lens of parents’ experiences within the diagnostic process, and how they adapt to their child’s diagnosis.

### 4.1. Parents Seek Diagnosis

The experience of parenting a child with ADHD is well-documented, with findings typically highlighting the significant challenges of the experience, such as exhaustion [34], interpersonal conflict [35], and powerlessness and invalidation to have your experience acknowledged by others [25]. Having a child with ADHD is associated with more parental stress [36] and psychiatric distress [37], the latter [37] being linked to the child’s behaviour problems. Clearly, studies with parents post-diagnosis suggest that rather than suddenly appearing, the diagnosis emerges and is often characterised by distress and significant challenges in daily life.

Therefore, while some diagnoses might be sudden or abrupt, an ADHD diagnosis might be unique, where the assumptive world has been significantly reconstructed long before diagnosis. As discussed in Nicholson and Lee [38], many parents of ADHD children are made aware of their children’s difficulties long before they begin the process of medical assessment and diagnosis. Parents often recount examples of how their children were doing something “different” or atypical at much younger ages than the typical diagnosis. The median age of first diagnosis is estimated to be between 7 and 9 years of age [39], which is typically when diagnosis in the UK and Ireland occurs. Typically, ADHD-related difficulties emerge during the very early years, including the challenges of family conflict and of meeting expectations at home, but also an increased risk of physical injuries and suspension or expulsion from pre-school [34,40,41,42].

This early recognition of difference in their children leads parents to begin a process of investigation and exploration, in which they collect evidence to quantify their concerns and begin recontextualising their child as needing a different approach or additional support [43]. While increased parental involvement reportedly does not necessarily reduce the negative impact of ADHD on academic difficulties [44], it is clear that parental pre-diagnostic awareness of their children’s differing needs and an increased involvement in education indicates changes to the assumptive processes of parents towards their children with ADHD occurring long before diagnosis.

We can also see pre-diagnostic meaning-making in studies of parental beliefs involved in making referrals for diagnosis. Hamed et al.’s [45] review of “barriers” to diagnosis claimed that parents must believe their child has a neurobehavioral disorder, that ADHD is a valid diagnosis, and that their child’s behaviour is not caused by other factors, such as diet or school. Pre-diagnostically, a parent must view their child’s behaviour as a problem, and as burdensome and stressful. However, certain presentations (e.g., inattentive ADHD) are less burdensome and stressful, and different cultures and different parents (mothers vs. fathers) have different behavioural expectations and tolerance for stress. We can acknowledge that these beliefs are necessary pre-conditions to seeking a diagnosis, while, at the same time, questioning whether they are barriers or problems. One could argue that such parental beliefs involve assumptive worlds where their child is different, and that is not only acceptable, but it is also positive. If so, then grief theory might not help us understand these parental/familial experiences, because those individuals do not have to cope with loss.

However, evidence suggests that societal reactions to the differences associated with ADHD are often negative. A growing body of research demonstrates that ADHD, like other mental health disorders, comes with a lot of social stigma, the effects of which add significantly to the difficulties people experience with daily life [46,47]. Many factors contribute to this stigma, such as negative media portrayals and public concerns about the validity of the diagnosis and treatment of ADHD, especially its medical management. Public understanding often does not appreciate the complexities involved and tends to trivialise the impact of ADHD, suggesting difficulties are caused by a lack of willpower or a resistance to conform [47,48,49].

Some evidence suggests that, pre-diagnostically, parental concern over societal stigmatisation and a desire not to label their child may lead some to avoid pursuing assessment until much later, which we can understand as engaging in a process of denial. Susan dosReis et al. [50] interviewed 48 parents about their experiences leading up to a diagnosis of ADHD. They said that parents reported experiences of stigma and initial fears about labels, as a result of societal and media constructions of ADHD and mistrust of ADHD and its treatment. While parents might initially deny the problem, and fear stigma and labels, their experiences of their child’s difficulties, isolation, rejection, and feeling dismissed can mean they actively seek a diagnosis [25,43,45,50]. In this way, we could understand the pre-diagnosis stage and its resolution as the recognition that their child’s development or behaviour differs from the “norm” and that there might be a “problem”, and then actively seek a diagnosis. For these parents, at least in part, the reconstruction of a new assumptive world (and accepting the potential presence of neurodivergence/disorder) may come long before diagnosis.

### 4.2. Obtaining an Assessment—Adjusting Expectations?

Once parents have adjusted their perception of their children’s difficulties to include the potential of a neurodivergent explanation, this begins the process of exploring this new assumptive world and gaining clarity, including confirming these assumptions. Parents begin to recognise their child’s development or behaviour is “different” or that something is “wrong” through medical and/or educational assessment. This process, conceptualised in the literature as the parental “Diagnostic Quest” [38,51], if often a lengthy and challenging journey, with many finding their difficulties invalidated and minimised by the professionals (in particular by gatekeepers to diagnostic pathways, such as teachers or general practitioners [GP]). Parents often feel forced to “fight” for their concerns to be recognised in the face of professional rejection and comments questioning their parental capacity and ability [25,38,50,51]. For example, recently in the UK, the waiting time for a child’s ADHD assessment was found to be around 4–6 years [52], meaning that parents have a protracted amount of time in which to adjust and solidify their assumptions of what their children’s needs may be and who their children are. However, this waiting time does not include the periods of “watchful waiting” recommended by NICE guidelines when children present in primary care facilities [53] nor the often-repeated rejections for assessments that they face [54].

During this time, many parents often begin to develop their knowledge by exploring ADHD-related literature and online content, constructing a coherent explanation of their children’s presentation, which incorporates an ADHD-related conceptualisation [43]. This process of knowledge development may well begin the process of recontextualising the parental view of their child from “typical child” to “potentially ADHD” child as they consume ADHD-related content which often mirrors or explains their children’s behaviour and enables them to make sense of their difficulties.

### 4.3. Post-Diagnostically Recontextualising the Child

Once a diagnosis is obtained, parents recontextualise their pre-existing beliefs and their idea of who their children are by developing new “self-understanding of the possibility for understanding and supporting their children” [55] (p. 6) in the face of a diagnostic label.

These post-diagnostic reactions can be framed both positively and negatively. In the former, parents may experience empowerment and/or relief following a diagnosis, which would enable a greater understanding of their child’s difficulties and provide access to resources, such as support groups and information [25,35]. An ADHD diagnosis allows one to be perceived not as a “problem person” but a “person who has a problem” [45], or to recategorize themselves from an “abnormal, normal person” to a “normal neurodivergent one” [38]. This crossing of a threshold, or “border crossing” [56], allows a dramatic adaptive turn in which a valid and clear explanation of their children’s needs is presented. These positive recontextualizations of children’s identity have been emerging more recently, challenging the narrative that receiving a medical diagnosis does not need to result in an undesirable, negative, and grief-laden process. Within lay populations, the development of the neurodiversity (or neurodivergence) paradigm has challenged pre-conceived notions of what it means to have a neurodevelopmental condition. The “tragedy” and undesirability of an ADHD diagnosis has been transformed into a more nuanced perspective, in which neurodivergence is being seen as having both negative and positive connotations [43]. The changing landscape of parental reactions to an ADHD diagnosis highlights this societal shift, as the medical discourse which prioritises impairment and disorder proves insufficient at capturing the totality of the parental experience. Therefore, one might ask, is grief theory more helpful in understanding the meaning-making process pre-diagnosis?

Although these more positive reimaginings of children post-diagnosis are becoming more commonplace, evidence tends to be collected for parents retrospectively (some time after a diagnosis, where a reconstruction of the assumptive world might have already occurred). Research also suggests more negative reframing in the face of diagnosis. Parents have expressed concerns over stigma and society labelling their child following negative media portrayals [50] as well as distress at having to incorporate navigating ADHD in their “new lives” [56], and worry about their child’s future [35,50]. Some parents go from being the parent of a “normal child” to the parent of a “disabled” child [56], which rocks the normality of their experience and disrupts the pre-conceived parental narrative trajectory [55]. They may also experience shame and guilt about their parenting, for example, for their parenting practices, their child’s struggles, or delays in diagnosis [25,43]. The diagnosis also gives rise to new fears, such as the fear of “zombification” [57] due to medication or the fear of a new conduit in which their children can be marginalised, discriminated against, and stigmatised [43]. Systematic reviews consistently [58,59] report the impact of stigma on parents’ decision-making about ADHD treatment. As such, at least for some parents, there is still some grief work to do post-diagnostically, as reality sets in and they must navigate their new lives, cope with guilt over the past and worries about their child’s future, and make decisions around treatment.

Interestingly, parental grieving or a negative reaction to a child’s ADHD diagnosis appears to be relative to the assumptive world of the parent pre-diagnostically. According to researchers, parents with a stronger pre-diagnostic conviction of their children’s ADHD or neurodivergent status may have less disruptive reactions to a diagnosis, leading to a cohesive experience in which their “pre-existing illness narratives” are confirmed, instead of being grief reactions [38]. Where their children experience significant ADHD-related difficulties, a diagnosis may be met with optimism, as parents hope teachers and other professionals might be better able to understand and support their child [35,60].

These competing conceptual paradigms of ADHD (i.e., neurodiversity and biomedical) present a unique meaning-making process relatively unexplored in the literature. Within Ringers’ [56] work, children diagnosed with ADHD are said to find the experience of diagnosis to come with competing, almost paradoxical, perspectives. On the one hand, they must adjust to environments and utilise strategies in order to cope, whilst also wanting others to accept them for “who they are”. Social supports such as family and friends are presented as useful in their ability to help, but also as a significant source of the demands which lead them to experience disability. Similarly, Ghosh et al. [35] found shifting, often conflicting narratives. The researchers report positive traits were being identified, and that at the same time, ADHD was viewed as an impairment to be fixed and that people needed to fit in and function in society.

For parents, similar competing perspectives arise post-diagnosis, as they battle with the competing narratives of a positive neurodiversity paradigm and a biomedical disorder one. Parents are said to be typically drawn to more strength-based neurodiversity paradigms [43,61], whilst struggling to navigate the theoretical tension [38] between these seemingly competing paradigms as they are “forced” through a medical process to diagnosis. They may see their children’s ADHD as a strength or even “superpower”, whilst also accepting a label which includes both “deficit” and “disorder”. A relatively common predicament relates to medication. ADHD assessment is typically followed by the use of a stimulant medication to reduce symptomology and impairment [62]; however, the use of medication seems to challenge some of the tenets of a neurodiversity perspective.

Overall, the parental meaning-making response to a child’s ADHD diagnosis does not begin at the time of diagnosis. It is a prolonged process, often beginning in early childhood, in which parents develop their assumptive world to construct an idea of their children, which very often includes an ADHD conceptualisation well before confirmation through medical diagnosis. The pre-diagnostic development of an assumptive world appears to have a direct relationship to whether the post-diagnostic parental experience of an ADHD diagnosis is one of grief, relief, or celebration.

## 5. Diagnosis in Adulthood: Adult Grief Work

We have explored diagnosis and parental meaning-making regarding an ADHD diagnosis for their child. However, with the advent of the DSM5 and subsequent increases in diagnosis in adulthood [33] an understanding of adults adjusting to their own diagnosis is needed.

### 5.1. Adults Seek Diagnosis

While there is limited research exploring pre-diagnostic meaning-making, post-diagnosis research with adults with ADHD highlights significant stress and significant challenges in daily life. Adult ADHD is associated with more relationship difficulties, family dysfunction, and conflict; higher rates of academic failure and school/college dropout; lower job performance and rank; more unemployment and underemployment; and higher levels of criminality [63,64]. An estimated 87.5% experience psychological distress, such as substance abuse/dependence, anxiety, and depression [64,65]. Population-based studies, where the individuals might not have received a diagnosis, provide further support for the pre-diagnosis stage being characterised by distress and challenges in navigating work, relationships, and daily responsibilities. Studies which estimate the prevalence of ADHD symptoms by screening adults attending out-patient mental health services across Europe, seeking support for other conditions, consistently identify ADHD symptoms within these populations [66].

Some evidence from the pre-diagnosis stage suggests that the impact of ADHD, especially undiagnosed ADHD, is “overwhelmingly chaotic” [26]. One study of European women with ADHD [67] found that women said their ADHD related difficulties caused significant distress and internalising problems, with others (e.g., [68,69]) suggesting other coexisting diagnoses may be blamed for the difficulties they experience. Indeed, the so-called “emotional toll” of ADHD includes these delays in diagnosis (e.g., [70]). Therefore, just as in childhood, seeking a diagnosis in adulthood does not emerge within a vacuum, it arises in the context of stress and difficulties in day-to-day life, where people recognise there is a “problem” with daily life, and seek support (including a diagnosis).

The literature also suggests that pre-diagnostic meaning-making is happening for adults, usually starting in childhood. Research consistently finds that adults with ADHD who are diagnosed in adulthood report that they felt “different” from a young age [68,69,71,72,73,74]. They describe having to work through ongoing experiences of marginalisation and “being different” through childhood and into their adult lives. As a result of these experiences, they tend to attribute the blame to themselves, and report viewing themselves as being personally “flawed”. They are said to develop low self-esteem and a poor sense of self [68,69,71,72,74]. Baig and Khaya [68] reported that adults said it was this feeling of being “broken” that prompted them to seek a diagnosis.

Diagnoses for adults may come after their own child receives a diagnosis. Adults report recognising the difficulties their child faces in their own experiences, and they tend to develop a lot of ADHD knowledge through their child’s diagnostic process. As a result, they might seek out a diagnosis for themselves (e.g., [25,43,69,75]). Indeed, the role of ADHD knowledge and stigma also appears to be important in the pre-diagnostic phase for adults with ADHD (e.g., [69]). For example, Baig and Kahya [68] report that women actively seek explanations for their difficulties, and, in doing so, develop a lot of ADHD knowledge. With this knowledge, the women said, they were able to self-advocate and fight for a diagnosis. However, the nature of that information matters. Just like in childhood, stigma associated with ADHD is also reported by adults as a barrier to diagnosis, and as associated with feelings of incompetency or inadequacy (e.g., [69,75]). In one review, Lebowitz [76] concluded that, while stigma varies between ADHD in childhood and adulthood, negative perceptions toward people with ADHD appear to generally be present at all stages of development.

While there may be a significant amount of meaning-making involved in the decision to seek a diagnosis, the difficulty does not end there, with adults reporting feeling misunderstood and dismissed by professionals when seeking support, and/or having to “fight” for a diagnosis [26,67,68]. Similarly, for example, Matheson et al. [26] reported that adults (N = 30) in the UK had an “uphill struggle” accessing diagnosis, and that delays in diagnosis contributed to or exacerbated other mental health or functioning problems, especially if their diagnosis was significantly delayed.

While there is limited research into the perspectives and experiences of adults regarding the pre-diagnosis stage, we can clearly see that their lived experiences are often marred by distress and challenges in daily life. It is reasonable to assume that, as a result of these negative experiences, they sought a diagnosis. In both childhood and adulthood, individuals need to recognise some degree of problematic difference in order to seek support and diagnosis. Otherwise, why would one seek a diagnosis? Accordingly, we could ask the question, is grief theory helpful to understand the experiences of those with more severe presentations of ADHD? The majority of those identifying as ADHD do not have a diagnosis [24]. However, it is equally plausible that many of these individuals are languishing on waiting lists struggling to obtain access to services. The role of ADHD knowledge, social support, and social stigma appear to be important factors (for better or worse) in the pre-diagnostic meaning-making process.

### 5.2. Post-Diagnostic Adjustment—Grieving and Reconstructing

Once they receive a diagnosis, findings are conflicting about adults’ immediate reaction to an ADHD diagnosis. Some research suggests the initial reaction adheres to an adjustment process similar to grief, where adults experience a period of denial. This is followed by turmoil, where they feel anger or sadness about their past, anxious about their future and their identity, and then, through a process of adjustment and meaning-making, they come to acceptance [77,78]. Other studies report relief and elation immediately following diagnosis; however, the process still reflects much of the cycle within grief theory, where diagnosis represented a significant psychosocial transition. After the elation passes, they experience confusion and emotional turmoil focused on their past and their future, including the need to incorporate the diagnosis into their identity [71,79,80].

The different initial responses reported in these studies, between denial (e.g., [77,78]) and elation/relief [79,80] might reflect meaning-making in the pre-diagnosis stage. Like parents, adults also report engaging in information gathering (e.g., [68]) and they may adopt a neurodivergent identity long before diagnosis (e.g., [24]). Indeed, Aoki et al. [77] reported that alongside denial, participants reported a lack of knowledge of ADHD and prevalent self-stigmatising beliefs; and over time, those adults did report a sense of relief. Adults report feeling relief because a diagnosis provides an explanation and legitimises the difficulties and histories of “being different”. This relief might be experienced immediately after diagnosis (e.g., [81]) or sometime later [77], depending on where the individual is in the meaning-making process. Clearly, further research into the pre-diagnosis stage and its impact on the post-diagnosis stage is required for adults with ADHD.

The Dual Process Model [4] of grief provides a useful framework to understand what is going on within the grief cycles of the meaning-making process experienced by adults post-diagnosis. There appear to be two central phenomena involved in adapting to the diagnosis.

First, adults may grieve over their past experiences and the child they were. This tendency to focus on memories is commonly found within the literature on grief. The ADHD literature suggests that regardless of whether the adult’s initial reaction is positive (e.g., relief) or negative (e.g., denial), it is followed by a period of confusion and turmoil. During this period, a range of emotions are experienced, especially anger and sadness [26,78,79,81]. Adults reflect on a lifetime of experiences and reframe their past in light of the diagnosis. As they reframe their past, they can experience anger and sadness. They might wish they had been diagnosed earlier and might believe their lives could have been different, where past failures in school/work could have been avoided and relationships could have been saved. They report feeling sad about the accumulated psychosocial burden after a lifetime of failure and missed potential.

The second phenomenon within grief theory is referred to as “restorative-oriented” behaviour or coping, where the individual adapts to changes and new roles and works towards rebuilding a new life. Post-diagnostically, adults reportedly focus on constructing and/or reconstructing a new identity. After a period of elation/denial and then grief, adults might encounter concerns around the diagnosis, such as wondering whether ADHD is a disorder or a gift, they might question whether ADHD is “real”, or have to figure out what ADHD means in terms of their identity (e.g., [81]). In Rogers’ [82] book *On Becoming a Person*, the chapter “This Is Me” begins “I speak as a person, from a context of personal experience and personal learning…” (p. 4). Our identity, therefore, encompasses the totality of all our beliefs (which are constructed through experience), that then determine how we view the world and events, and it helps guide our actions. Identity is constructed (and reconstructed) socially through interactions [83]; the process is heavily reliant on symbols (e.g., social norms, behavioural expectations) and autobiographical memories [84]. A key part of the psychosocial adaptation involved in receiving a diagnosis is what it means for one’s identity.

Evidence suggests that negotiating the meaning-making post-diagnosis includes considering identity both pre- and post-diagnosis. Identity before diagnosis involves adults reflecting on the inappropriate or incorrect labels they received in childhood, such as “lazy”, “bold”, or “depressed” [67,77,78], which could result in lower self-worth [78]. Post-diagnostically, adults have the opportunity to reframe these experiences and labels with a new lens, providing explanations for difficulties [77,78,79] and opportunities for greater self-worth and reduced self-blame [78]. However, several studies also suggest that post-diagnostic identity reconstruction includes concerns, where adults may be anxious about what a diagnosis means for their future, and about its permanency, the idea that ADHD is here to stay [78,79,81]. Young et al. [79] reported adults had to cope with these fears and anxieties about their future before being able to accept their new realities and finding positives.

Several research studies report the final stages of the process involve acceptance and “finding the positives” in ADHD and/or their experiences [35,78,79]. The neurodiversity paradigm does provide a supportive lens within the reconstruction of the assumptive world (particularly around identity and meaning); because it includes a strengths-based understanding of ADHD and argues there should not be a “normal”. In this way, the neurodiversity paradigm supports the meaning-making process (especially understanding the past and reconstructing identity), where people adjust to the realisation that something (e.g., behaviour) is different, and neurodiversity explains/normalises this difference, while at the same time providing strengths and celebrating aspects of this diversity. This may happen pre- and/or post-diagnosis, depending on when this realisation happens.

The reconstruction of identity is a complex process, and adults may experience difficulties constructing a coherent identity after their diagnosis (e.g., [85]). Within this process of self-understanding, the adult must navigate multiple potential identities, which parents do for their child in childhood. Adults have access to positive recontextualizations of their identity within a neurodiversity paradigm, suggesting that diagnosis does not necessarily need to be an undesirable, negative, and grief-laden process. Research has identified many positive aspects of adult ADHD, such as energy, creativity, hyperfocus, nonconformism, adventurousness, and sublimation [86]. For those who do seek and receive a diagnosis, findings from Carr-Fanning [67] suggest that adults report struggling to navigate multiple conflicting identities. On the one hand, women with ADHD reported the diagnosis as empowering, and a desire to lean in to their neurodivergent identity. While, on the other hand, this framework may not reflect their actual lived experiences of being disabled; that is, chronic failure and rejection, the need for support and accommodation, and using medication to treat their deficits. These shifting narratives and conflicting frameworks are reported elsewhere [35,81]. Ghosh et al. [35] found that adults identified positives of ADHD, while also acknowledging that it was an impairment to be fixed. French and Cassidy [87] suggested that the framework does not matter, and positive post-diagnostic change and acceptance come from the “understanding” one gains through a diagnosis. How the person makes sense, however, is significant, because Ghosh et al. [35] reports that adults’ and parents’ beliefs impacted the treatment choice. Tal and Goodman [73] found that pre-diagnostic meaning-making was important here. They said that not receiving a diagnosis until adulthood provides people with an opportunity to “invent personal methods for handling a problem” ([73] p. 202), which may impact treatment choices post-diagnosis (e.g., reduce reliance on medical management).

## 6. Discussion and Conclusions

This paper explored whether and how grief theory can support an understanding of the process of adapting to a diagnosis for both parents and adults. The analysis suggests that grief models, particularly meaning-making approaches [5,28] and the Dual Process Model [4], provide valuable frameworks for conceptualising the emotional, cognitive, and social processes of adjustment across the diagnostic journey. Unlike bereavement following a death, where the loss is typically acute and identifiable, grief in the context of ADHD is anticipatory, cumulative, and often unfolds across developmental timeframes. Parents may begin grieving pre-diagnosis when noticing developmental or behavioural differences in their child [1], while adults may experience retrospective grief for the child they once were and the opportunities they perceive as missed [27]. In both cases, the diagnosis represents not an endpoint but a psychosocial transition that requires a reconstruction of meaning, identity, and life narratives.

The Dual Process Model [4] is particularly instructive here, as it highlights the oscillation between loss-oriented coping (e.g., grieving for an expected child, regretting lost years) and restoration-oriented coping (e.g., developing new parental roles, constructing an ADHD-informed identity). This oscillation captures the past- and future-focused elements of adjustment more effectively than earlier stage-based models [3], which have been critiqued for their linearity and limited capacity to represent individual variability (e.g., [4]). Meaning-making perspectives similarly frame the diagnostic process as requiring the reconstruction of a coherent narrative that integrates altered expectations into a revised “assumptive world” [1,28]. For parents, this may involve reshaping their understanding of their child’s future, while for adults, it may entail reinterpreting their past and re-establishing a sense of continuity in light of new self-knowledge [5].

Importantly, the findings also indicate that the pre-diagnostic phase is not neutral: grief work may begin well before a formal diagnosis is received. Parents and adults often engage in anticipatory meaning-making when they first recognise divergences from expected developmental trajectories [28]. These processes are influenced by available information and by exposure to societal stigma, which shape whether diagnosis is experienced as relief, validation, or distress [29]. For some, a coherent pre-diagnostic construction of ADHD supports positive adjustment, whereas for others, conflicting cultural framings of neurodiversity and disability may intensify grief and complicate adaptation.

Clinical and practical implications arise from applying grief theory to this context. Recognising that grief can begin before diagnosis underscores the importance of accessible, neuro-affirmative, evidence-based information at the pre-diagnostic stage, including for those on waiting lists or considering referral. Post-diagnosis, support should extend beyond biomedical explanation, toward facilitating psychosocial adjustment, identity reconstruction, and meaning-making. Professionals need to be attentive to the tensions between strengths-based discourses, which may foster resilience and acceptance, and recognition of lived disability, which validates experiences of difficulty and informs support and treatment decisions. However, neuro-affirmative paradigms may also undermine lived experiences of marginalisation and confusion within identity reconstruction and the new assumptive world. Helping individuals and families navigate these tensions may reduce the risk of unresolved grief, which has been associated with difficulties in generating coherent narratives of loss and adaptation [5].

In conclusion, grief theory provides a useful lens for understanding the complexities of adapting to an ADHD diagnosis for both parents and adults. By moving beyond linear or prescriptive models toward frameworks that emphasise oscillation, narrative reconstruction, and meaning-making, we can better appreciate the heterogeneity of experiences surrounding diagnosis. Parents may grieve altered futures for their children, while adults may grieve lost years of misrecognition; both, however, must reconstruct assumptive worlds that accommodate new realities. Conceptualising diagnosis as a grief-related process not only advances theoretical understanding but also carries significant implications for practice. Diagnostic and post-diagnostic support should anticipate emotional turbulence, address stigma, and facilitate the development of resilient and coherent identities.

## 7. Future Directions

Clearly, there is a lack of clarity around perceptions, experiences, and meaning-making within the pre-diagnostic stage, especially for adults. It would be useful to understand this stage better, and to identify and explore particular experiences or framings of a diagnosis/neurodivergence, and the impact of these on psychosocial adjustment in the post-diagnosis stages. Future research might consider the following questions:

- What personal and social factors contribute to adults’ and parents’ decision to seek a diagnosis?
- What personal and social factors contribute to adults’ decisions not to seek a diagnosis and to self-identify as ADHD?
- What frameworks do adults and parents hold about ADHD prior to diagnosis?
- What pre-diagnostic frameworks and experiences enable more positive experiences and adjustments post-diagnosis?

The literature also raises important questions about the tensions in diagnostic work posed by competing paradigms (i.e., neurodiverse vs. biomedical). This paper has highlighted the dissonance between the two dominant and competing frameworks. Clearly, there are differences in reactions to receiving a diagnosis (e.g., denial vs. relief), especially for those who do not already hold a pre-existing neurodiversity framework. We can understand the experience of relief as the individual being farther along in the psychosocial adaptation process and/or the adoption of a neurodiversity framework. However, in theory, the neurodiversity framework (or the acceptance and celebration of difference) could reduce the need, requirement, or effectiveness of a grief theory lens. In a similar line of questioning, we do not know about the perspectives and experiences of the significant number of adults who chose to self-identify as neurodivergent without seeking a formal diagnosis. Future research should explore how, when, and why the neurodiversity paradigm is adopted by people with and without a formal diagnosis to better understand how the framework informs psychosocial adaptation. We also need to understand if and when the neurodiversity framework is not appropriate. Future research could explore the following questions:

- How, when, and why do neurodivergent adults and parents of neurodivergent children use the neurodiversity paradigm?
- How does the neurodiversity paradigm support psychosocial adaptation in children and adults who receive a diagnosis?
- How do parents and/or adults adopt the neurodiversity paradigm and live without a formal diagnosis?
- What are the difficulties with and barriers to adopting the neuro-affirmative paradigm in childhood and adulthood?

In the exploration of both of the above areas, it is necessary to consider the ADHD presentation (e.g., hyperactive-impulsive vs. inattentive) and its severity, as well as co-existing diagnoses. Presentation and severity will affect the degree of distress and difficulties the person and their family experience. Certain presentations may be more difficult to fully accommodate within neuro-affirmative frameworks, and may be more associated with stress, loss, and so grief.

The lived experience of ADHD and the seeking of and adapting to diagnoses will vary across countries and groups, because many of the processes involved in psychosocial adaptation, meaning-making, and behavioural expectations are socio-culturally embedded. Therefore, a significant limitation within the existing literature is that the majority of the research is in contexts that (and with participants who) are white, Western, and Eurocentric. There are some exceptions, most notably the Japanese study by Aoki et al. [77]. More research is required to understand the perspectives and experiences of diagnosis in non-Western contexts and with members of multi-ethnic communities.

## Abbreviation

The following abbreviation is used in this manuscript:

ADHD	Attention Deficit Hyperactivity Disorder
